# Exploring the item sets of the Recovering Quality of Life (ReQoL) measures using factor analysis

**DOI:** 10.1007/s11136-018-2091-1

**Published:** 2018-12-21

**Authors:** Anju Devianee Keetharuth, Jakob Bue Bjorner, Michael Barkham, John Browne, Tim Croudace, John Brazier

**Affiliations:** 1 School of Health of Related Research, University of Sheffield, Sheffield, UK; 2 Optum, Johnston, RI USA; 3 University of Copenhagen, Copenhagen, Denmark; 4 Department of Psychology, University of Sheffield, Sheffield, UK; 5 University College Cork, Cork, Ireland; 6 Dundee Centre for Health and Related Research, University of Dundee, Dundee, UK

**Keywords:** Recovering Quality of Life, Factor analysis, Bi-factor model, Dimensionality, Latent structure

## Abstract

**Purpose:**

This paper presents two studies exploring the latent structure of item sets used in the development of the Recovering Quality of Life mental health outcome measures: ReQoL-10 and ReQoL-20.

**Method:**

In study 1, 2262 participants completed an initial set of 61 items. In study 2, 4266 participants completed a reduced set of 40 items. Study 2 evaluated two formats of the questionnaires: one version where the items were intermingled and one where the positively worded and negatively worded items were presented as two separate blocks. Exploratory and confirmatory factor analyses were conducted on both datasets where models were specified using ordinal treatment of the item responses. Dimensionality based on the conceptual framework and methods effects reflecting the mixture of positively worded and negatively worded items were explored. Factor invariance was tested across the intermingled and block formats.

**Results:**

In both studies, a bi-factor model (study 1: RMSEA = 0.061; CFI = 0.954; study 2: RMSEA = 0.066; CFI = 0.971) with one general factor and two local factors (positively worded questions and negatively worded questions) was preferred. The loadings on the general factor were higher than on the two local factors suggesting that the ReQoL scale scores can be understood in terms of a general factor. Insignificant differences were found between the intermingled and block formats.

**Conclusions:**

The analyses confirmed that the ReQoL item sets are sufficiently unidimensional to proceed to item response theory analysis. The model was robust across different ordering of positive and negative items.

**Electronic supplementary material:**

The online version of this article (10.1007/s11136-018-2091-1) contains supplementary material, which is available to authorized users.

## Background

The Recovering Quality of Life (ReQoL) is a self-report instrument to measure health outcomes for people with mental health difficulties [1]. Two versions of the measures, ReQoL-10 and ReQoL-20, have been constructed for use in routine practice as well as in research including clinical trials. They are self-report measures suitable for use by individuals aged 16 and over experiencing a wide spectrum of mental health conditions and levels of severity. Traditionally, mental health outcomes have tended to be symptom-based rather than reflecting the service users’ recovery in their quality of life. While there are measures focusing on the process of recovery [2], a recent review identified the need for a patient-reported outcome measure (PROM) that measures the outcomes of recovery in terms of those aspects of quality of life that matter to mental health service users [3]. Hence the ReQoL measures were developed. The initial psychometric analysis of the measures demonstrated acceptable scaling properties, reliability, validity and responsiveness [1].

**Fig. 1:**
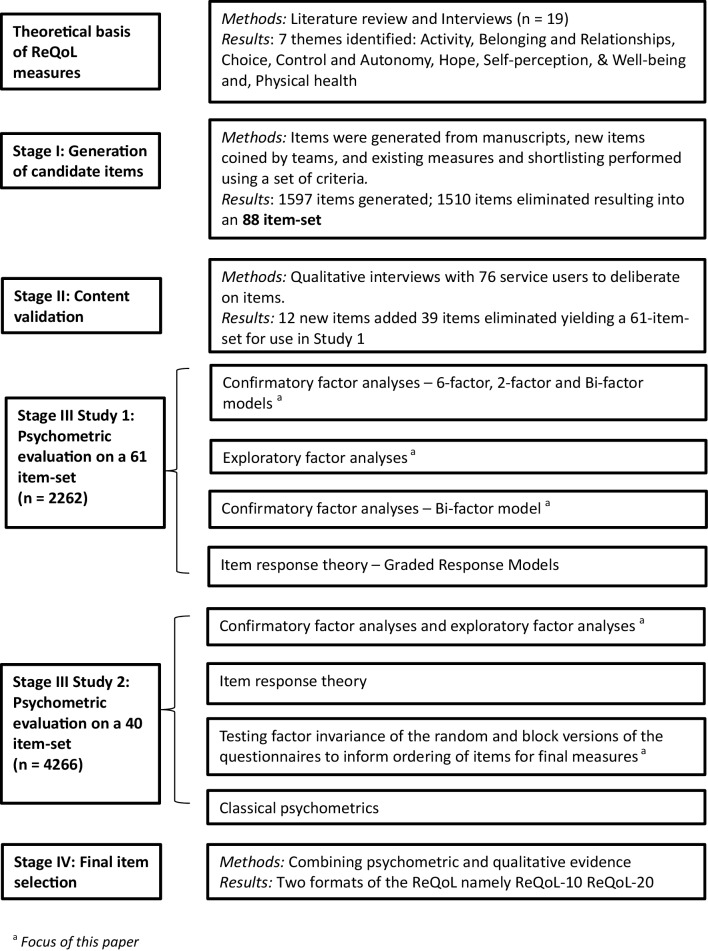
Development process of the ReQoL

The two ReQoL measures tap seven domains that are relevant to the recovery of people with mental health issues: activity (meaningful and/or structured); belonging and relationships; choice, control and autonomy; hope; self-perception; well-being and physical health. These domains were identified in a systematic review of qualitative research on Quality of Life (QoL) in mental health and from interviews with service users [4–6]. Service users reported both positive and negative aspects of the themes (e.g. hope/hopelessness), which either enhanced or diminished their quality of life. Connell et al. [6] presented the justification that it was important for both positive and negative aspects to be reflected in any QoL measure in mental health. Similar themes had been found elsewhere in the literature. Leamy et al. [7] identified the following themes which map onto the ReQoL themes identified in parentheses: connectedness (belonging and relationships), hope (hope), identity (self-perception), meaning (meaningful activity) and empowerment (choice, control and autonomy). Therefore, the six mental health themes and the one physical theme formed the conceptual basis for the ReQoL measures (Fig. 1).

The items used in the ReQoL measures were generated in two stages. In stage I, items were adapted from existing measures, from qualitative interview manuscripts and new items were also generated. In all cases, the criteria suggested by Streiner and Norman were used to shortlist and refine items to enhance comprehension and avoid misinterpretation [8]. In stage II, a total of 88 items were presented to 76 service users who were asked to assess their suitability. This produced 61 items that were used in subsequent psychometric analyses [9].

In this paper, we present the results of factor analyses carried out to establish the latent structure of the ReQoL item sets that would be used to construct the final ReQoL measures. This was an important step for the following reasons. First, establishing a reproducible understanding of the latent structure of the ReQoL items addresses internal construct validity—the degree to which the measure assesses the themes of interest—as well as facilitates a higher fidelity and more accurate perspective when it comes to external scale validation [9]. Second, assessment of dimensionality is important for informing the scoring procedures. Third, an assumption of sufficient unidimensionality typically needs to be satisfied before undertaking item response theory (IRT) model estimation for the purpose of item reduction. Furthermore, unidimensionality is useful for interpreting statistics such as internal consistency. Accordingly, the main aim of this paper is to describe results regarding the factorial structure of the ReQoL item sets.

Mental health questionnaires often report a factor solution involving two correlated factors: positive mental health and negative mental health [10]. It is unclear to what extent such two-factor structure reflects a clear separation of positive and negative mental health or rather a methodological artefact of intermingling positive and negative items. With such intermingling, careless answering would result in a two-factor structure, even in the case of a unidimensional mental health domain. Thus, a secondary aim of the paper is to investigate the impact of the ordering of positively worded and the negatively worded items on the factor structure.

## Methods

### Study participants

Two sequential studies were conducted. In study 1, 1762 participants were recruited from four different sources: (i) 13 provider organisations, (ii) 3 GP surgeries (*n* = 3), (iii) 2 charities and (iv) from a trial cohort. An additional further 520 participants were recruited from an online panel, 200 of whom that had no mental health problem(s). As shown in Table 1, the total number of participants recruited in study 1 was 2262. In study 2, 4266 participants were recruited in the following broad diagnostic groups: depression, anxiety, psychotic disorders, bipolar disorders, personality disorders, eating disorders and others (severity ranged from mild to severe). Participants in study 2 were recruited from similar sources as in study 1; both samples are discussed in greater detail elsewhere [1]. The mean age of participants was 48 and 47 years, and the percentage rates of females were 58% and 55% in studies 1 and 2, respectively. Data were collected at one time-point only in study 1 but study 2 participants were followed-up between 6 and 12 weeks. In this paper, only baseline data are analysed from study 2. The raw scores and endorsement frequencies are presented in Supplementary Tables S1 and S2 showing the heterogeneity of the samples.

**Table 1:** Description of the samples

Sources	Study 1			Study 2		
	No. of recruiting organisations	No. of participants recruited (%)	Mean ReQoL-10 score (sd)	No. of recruiting organisations	No. of participants recruited (%)	Mean ReQoL-10 score (sd)
Provider organisations (NHS mental health trusts)	13	1352 (60%)	20.42 (9.65)	20	2862 (67%)	19.49 (9.72)
GP surgeries	3	145 (6%)	21.13 (9.08)	3	1146 (27%)	27.34 (9.63)
Charities	2	59 (3%)	20.41 (8.05)	3	45 (1%)	21.30 (9.48)
Trial cohort^a^	1	186 (8%)	23.52 (8.27)	1	213 (5%)	26.60 (7.81)
Online panel—service users	1	320 (14%)	19.21 (8.59)			
Online panel—no mental health problems	1	200 (9%)	29.64 (6.65)			
Total		2262			4266	

^a^This was a group of older people (aged > 65) who were originally not experiencing depression or low mood recruited as part of a trial and they also agreed to be contacted for related research as part of a cohort

### ReQoL item sets

Participants in study 1 were presented with 61 items (57 that address mental health and four addressing physical health). The aim was to reduce the number of items to 40 for presentation in a shorter questionnaire in subsequent study 2. The item reduction process combined insights from qualitative research and quantitative analysis and is discussed elsewhere [18]. The 40-item ReQoL item set presented in study 2 comprised 39 mental health questions and one physical health question including a mix of both positively worded (e.g. I felt happy) and negatively worded items (e.g. I felt unable to cope). The themes of the ReQoL and the number of items contributing to each theme in both items sets are described in Table 2. Participants responded on a frequency-based 5-point scale with the following verbal anchors for each ReQoL item: *none of the time, only occasionally, sometimes, often* and *most or all of the time*.

**Table 2:** Themes with positive and negative sub-themes with number of items

Themes	Positive sub-themes	Negative sub-themes
Activity	Enjoyable activity Meaningful/valued/purposeful/constructive (Study 1: *n* = 3; study 2: *n* = 2)	Unenjoyable/stressful activity Boring/meaningless/not valued (Study 1: *n* = 4; study 2: *n* = 3)
Belonging, relationship	Sense of belonging Positive relationships Friendship and camaraderie Supportive relationships (Study 1: *n* = 5; study 2: *n* = 2)	Not belonging—outsider Negative relationships Lack of friends Unsupportive relationships (Study 1: *n* = 4; study 2: *n* = 3)
Choice, control, autonomy	Autonomy Choice Control/coping (Study 1: *n* = 5; study 2: *n* = 3)	Dependence Lack of choice No control/not coping (Study 1: *n* = 2; study 2: *n* = 2)
Hope	Hope Plans and goals (Study 1: *n* = 2; study 2: *n* = 1)	Hopelessness Lack of plans and goals (Study 1: *n* = 4; study 2: *n* = 3)
Self-perception	Positive self-identity Positive self-confidence (Study 1: *n* = 4; study 2: *n* = 3)	Negative self-identity Negative self-confidence (Study 1: *n* = 6; study 2: *n* = 2)
Well-being	Happiness Relaxed/calm Positive energy/motivation (Study 1: *n* = 4; study 2: *n* = 4)	Depression/sadness Fear/anxiety/worry Loss of energy/motivation (Study 1: *n* = 14; study 2: *n* = 11)
Total mental health items	Study 1: *n* = 23; study 2: *n* = 1	Study 1: *n* = 34; study 2: *n* = 24
Physical health items	None	Study 1: *n* = 4; study 2: *n* = 1

### Presentation of the ReQoL items

Participants in study 2 were presented with one of the two formats of the item set: (i) a format where the positively worded and negatively worded questions were intermingled, that is not grouped in any way (henceforth referred to as format I) and (ii) a format where all the negatively worded questions were presented as a block followed by the positively worded questions (henceforth referred to as format B). If a two-factor structure is to some extent due to intermingling of positive and negative items, we would expect a clearer two-factor structure in the intermingled format than in the block format. Each recruiting organisation used only one of the two formats. A total of 2447 (from 16 recruiting organisations) and 1819 participants (from 12 organisations) completed format I and B, respectively.

### Statistical analyses

**Fig. 2:**
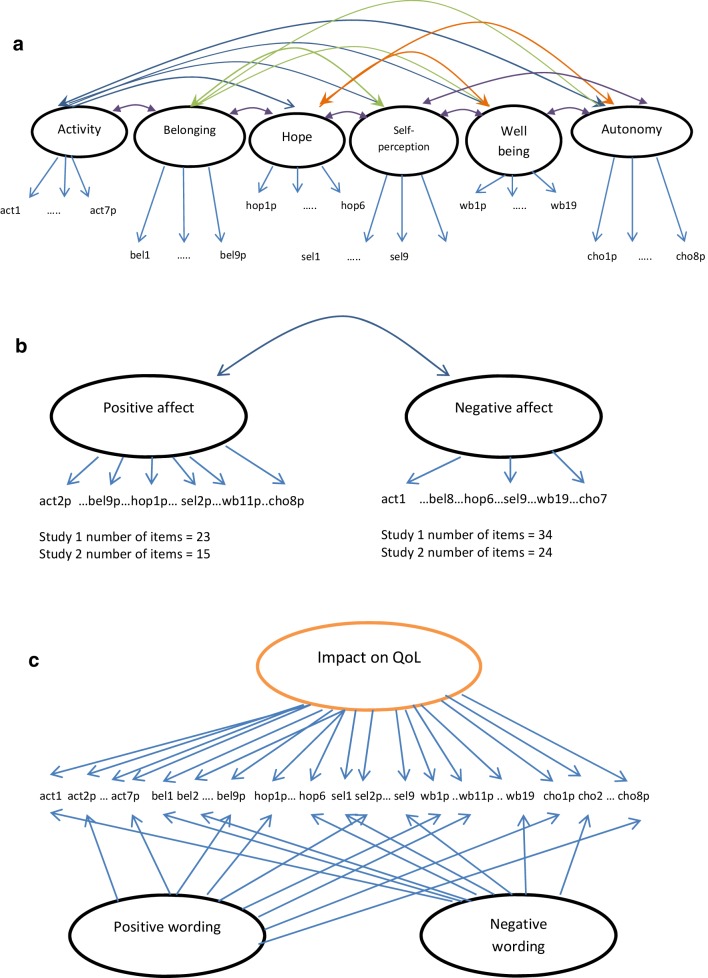
**a** Six-factor correlated traits model. **b** Two-factor correlated traits model. **c** Bi-factor model

The ReQoL was constructed using a conceptual framework comprising six mental health domains and a physical health domain [5, 6]. Therefore, confirmatory factor analysis (CFA) was undertaken first to test an a priori model consistent with the number of themes underpinning ReQoL item content. In Fig. 2a, this is shown diagrammatically, with items in each theme loading only on their respective (intended) latent factor and with all factors allowed to correlate.

Second, exploratory factor analysis (EFA) was also carried out using Geomin rotation to identify other potential factors present in the data. Finally, informed by the initial CFA and EFA, single factor, two-factor (Fig. 2b) and bi-factor (Fig. 2c) CFA models were estimated. A key advantage of CFA in this context is that it allows the comparison of competing models. Exploratory, confirmatory factor and bi-factor analyses were performed treating the items as ordinal categorical, using the robust-weighted least squares means and variance-adjusted (WLSMV) estimator [11] in Mplus 7.4 [12]. Model fit was assessed by the root mean square error of approximation (RMSEA) [13] and the comparative fit index (CFI) [14] where a value of ≤ 0.08 and > 0.95 was assumed to provide a good fit, respectively. In the bi-factor models that have all items loading on a common factor, we calculated the explained common variance for the global factor. This gauges the importance of the global factor relative to others [15]. The sequence of factor analysis models described above was then repeated in the study 2 dataset for the smaller item set of 40 items. To obtain finer factor solutions, residual correlations and modification indices (MI) were inspected to identify potentially redundant items [16, 17]. In the final models, local dependence between items (where the latent variables are not sufficient to explain the association between items) was introduced guided by:

(i) the highest MI (> 100) [17] or
(ii) whether the pairs of items had been identified as conceptually similar at the time of item generation and from qualitative evidence [18], by constraining the pair of items as free parameter estimates in model revisions, one at a time.

### Impact of ordering of items on the factorial structure

The design of study 2 enabled us to compare latent factor structure for datasets collected using different item ordering formats of the item set. We used multigroup CFA approach, where the persons answering the block item order constituted one group and the persons answering the intermingled format, the other group. We estimated a two-factor CFA model with the correlation between the two factors constrained to being equal in across groups and compared this to a model where this correlation was allowed to vary between groups. A significant result would indicate that the correlations between the positive and negative factors in the two formats were different which, in turn, would indicate that the ordering of the question impacted on the results. In these analyses, factor loading and item thresholds were constrained to be equal across groups. We evaluated global fit of the model, but since our research question was operationalized as a test of the equality of factor correlations, we did not test whether each item loading or item threshold differed between the groups. Finally, we sought to establish to what extent factor solutions suggested invariance across groups within studies and across samples [19].

## Results

### Initial confirmatory factor analyses results (studies 1 and 2)

Results and model fit statistics obtained for CFA factor solutions for both studies (study 1; study 2) are shown in Table 3. The hypothesised six-factor model did not achieve adequate fit (study 1: RMSEA = 0.087 CFI = 0.900; study 2: RMSEA = 0.095 CFI = 0.937). In study 1, the standardised factor loadings ranged from 0.493 (I had choices about what I did) to 0.903 (My life seemed pointless). The correlations between factors were very high ranging from 0.840 (belonging and well-being) to 0.954 (choice and activity). In study 2, the standardised factor loadings ranged from 0.645 (I had problems with my sleep) to 0.909 (I felt like a failure). The correlations between factors were very high ranging from 0.878 (activity and belonging) to 0.974 (choice and activity).

**Table 3:** Fit statistics from confirmatory factor analytic models (without model revisions)

	6-Factor model		2-Factor model: negative, positive		Bi-factor model: global, negative, positive	
	Study 1	Study 2	Study 1	Study 2	Study 1	Study 2
*χ*^2^ value	28,397	26,483	17,829	19,183	17,753	17,292
DF	1524	687	1538	701	342	663
RMSEA (< 0.08)	0.087	0.095	0.068	0.079	0.070	0.077
CFI (> 0.95)	0.900	0.937	0.940	0.955	0.940	0.960

### Exploratory factor analyses results (studies 1 and 2)

Eigenvalue analysis in (study 1, study 2) identified a strong first factor and a weak second factor as shown in Table 4 with eigenvalues for the first two factors at (31.4 and 3.4; 23.9 and 2.3). In the Geomin-rotated two-factor EFA solution, the first factor comprised the negatively worded items and the second factor the positively worded items. The inter-correlation between factors was .789 in study 1 and .80 in study 2.

**Table 4:** Eigenvalues from exploratory factor analysis

	Factor 1	Factor 2	Factor 3	Factor 4	Factor 5	Factor 6
Study 1—Eigenvalues	31.402	3.353	1.646	1.213	1.104	1.063
Study 2—Eigenvalues	23.901	2.501	2.501	1.093	0.838	0.795

### Estimating two-factor and bi-factor CFA models (studies 1 and 2)

The two-factor CFA model with the factors for positively worded items and negatively worded items returned the following model fit results: (study 1: RMSEA = 0.068 CFI = 0.940; study 2: RMSEA = 0.079 CFI = 0.955). The bi-factor model also had acceptable fit (RMSEA = 0.070 CFI = 0.940; RMSEA = 0.077 CFI = 0.960). ReQoL item loadings on the general/global factor were substantially higher than the two specific/group factors. Explained common variance (ECV) values were 78.9% in study 1 and 84.5% in study 2. (Tables S3, S4 in the supplementary materials show the factor loadings.)

### CFA models and modelling of local correlations (studies 1 and 2)

It was possible to identify and therefore extend our models to account for areas of strain within factor solutions, through local correlations. The model results are presented in Table 5. The two-factor models in both studies had very similar and acceptable fit (RMSEA = 0.066 CFI = 0.945; RMSEA = 0.064 CFI = 0.971). In study 1, the standardised factor loadings ranged from 0.495 (There were people I could turn to for help) to 0.896 (My life seemed pointless). The correlation between the two factors was .833. This fit was obtained after allowing three pairs of items to correlate with each other (see Supplementary Online Materials—Table S3). In study 2, the standardised factor loadings ranged from 0.656 (I had problems with my sleep) to 0.901 (I felt hopeless; Everything in my life felt bad). The correlation between the two factors was .835 (Table 5 for study 2 factor loadings).

**Table 5:** Fit statistics from confirmatory factor analytic models (with local correlations)

	2-Factor model: negative, positive		Bi-factor model: global, negative, positive	
	Study 1	Study 2	Study 1	Study 2
*χ*^2^ value	16,456	12,662	13,976	12,772
DF	1535	691	1596	653
RMSEA (< 0.08)	0.066	0.064	0.061	0.066
CFI (> 0.95)	0.945	0.971	0.954	0.971

The bi-factor models in both studies achieved acceptable fit (RMSEA = 0.061 CFI = 0.954; RMSEA = 0.066 CFI = 0.971) at very similar levels to the two-factor models. ECVs were 79.2% and 80.9% for general factors for study 1 and 2, respectively. In study 1, the standardised factor loadings for the 61-item set general factor were substantially higher than the two group/specific factors. The factor loadings of the general factor ranged from 0.424 (There were people I could turn to for help) to 0.892 (I felt confident in myself) while the factor loadings for the “negatively worded items-specific factor” ranged between 0.285 (I had problems with my sleep) and 0.518 (I felt panic), and 18 out of the 23 “positively worded items-specific factor” had loadings less than 0.3 (see Table S3). As shown in Table 6, in study 2, the factor loadings for the ReQoL general factor ranged from 0.571 (People around me cause me distress) to 0.873 (I felt confident in myself). The loadings for the “negatively worded items-specific factor” were between 0.300 (I found it difficult to get started with everyday task) and 0.483 (I felt terrified). 11 out of the 15 loadings on the “positively worded items-specific factor” were lower than 0.3.

**Table 6:** Parameter estimates for the two-factor and bi-factor models for study 2

Description		Two-factor model		Bi-factor model		
		Standardised loadings		Standardised loadings		
		Neg	Pos	Global	Neg	Pos
I found it difficult to get started with everyday tasks	ACT1	0.739		0.659	0.300	
I did things I found rewarding	ACT2P		0.803	0.733		0.458
I neglected myself	ACT3	0.764		0.669	0.363	
I avoided things I needed to do	ACT4	0.786		0.673	0.399	
I enjoyed what I did	ACT5P		0.866	0.815		0.350
People around me caused me distress	BEL1	0.68		0.571	0.389	
I felt lonely	BEL2	0.797		0.704	0.362	
I felt able to trust others	BEL3P		0.709	0.693		0.124
I felt people did not want to be around me	BEL4	0.796		0.698	0.378	
I thought people cared about me	BEL5P		0.684	0.640		0.306
I could do the things I wanted to do	CHO1P		0.763	0.723		0.296
I felt overwhelmed by my problems	CHO2	0.876		0.742	0.479	
I had the opportunity to do the things I wanted	CHO3P		0.731	0.671		0.418
I felt unable to cope	CHO4	0.894		0.772	0.454	
I felt in control of my life	CHO5P		0.884	0.861		0.183
I felt hopeful about my future	HOP1P		0.783	0.746		0.271
I felt hopeless	HOP2	0.901		0.790	0.429	
Everything in my life felt bad	HOP3	0.901		0.787	0.436	
I thought my life was not worth living	HOP4	0.836		0.734	0.394	
I felt like a failure	SEL1	0.89		0.786	0.408	
I felt confident in myself	SEL2P		0.892	0.873		0.162
I felt at ease with who I am	SEL3P		0.87	0.849		0.166
I valued myself as a person	SEL4P		0.875	0.855		0.160
I disliked myself	SEL5	0.872		0.796	0.327	
I felt calm	WB1P		0.812	0.803		0.038
I felt miserable	WB2	0.855		0.761	0.375	
I felt safe	WB3P		0.745	0.734		0.097
I was disturbed by unwanted thoughts and feelings	WB4	0.793		0.663	0.457	
I felt irritated	WB5	0.738		0.620	0.422	
I felt angry	WB6	0.72		0.600	0.424	
I felt relaxed	WB7P		0.865	0.847		0.115
I felt terrified	WB8	0.791		0.651	0.483	
I felt everything was an effort	WB9	0.821		0.716	0.397	
I felt panic	WB10	0.82		0.698	0.442	
I felt happy	WB11P		0.898	0.869	0.430	0.220
I found it hard to concentrate	WB12	0.807		0.689	0.441	
I worried too much	WB13	0.791		0.668	0.439	
I felt anxious	WB14	0.822		0.702	0.312	
I had problems with my sleep	WB15	0.656		0.575		

Residual correlations			
Two-factor model		Bi-factor model	
POS with NEG	0.836	Global with NEG	0
		Global with POS	0
		NEG with POS	0
wb6 with wb5	0.477	wb5 with wb6	0.455
bel5p with bel3p	0.384	bel5p with bel3p	0.401
wb13 with wb14	0.469	wb13 with wb14	0.450
wb10 with wb8	0.505	wb10 with wb8	0.477
wb14 with wb10	0.403	wb14 with wb10	0.384
wb9 with act1	0.308	wb9 with act1	0.338
sel3p with sel4p	0.318	sel3p with sel4p	0.344
cho1p with cho3p	0.374	cho1p with cho3p	0.299
bel4 with bel5p	0.288	bel4 with bel5p	0.315
wb8 with wb3p	0.289	wb8 with wb3p	0.339
		act4 with act1	0.250
		wb1p with wb7p	0.248

### Potential redundant items

In study 1, three pairs of items (total 5 items) and 12 pairs of items (total 21 items) were identified as potential local dependence residual correlations in the two-factor and bi-factor solutions, respectively. In study 2, 10 pairs of items (total 17 items) and 12 pairs of items (total 21 items) were identified as potential local dependence residual correlations in the two-factor and bi-factor models, respectively (see Table 6). These results, qualitative evidence and other quantitative evidence informed reduction of the item sets even further. These decisions are discussed more fully elsewhere [20].

### Comparing the format I and B of the 40 item set in terms of association between positive and negative item factors

The model fit for the intermingled and block formats was acceptable (RMSEA = 0.054 and CFI = 0.976). The common correlation of the positive and negative factors across formats was estimated at .829. A *χ*^2^ test for difference in correlations between factors across forms was not significant (*p* = .378). Thus the two formats did not differ.

## Discussion

We used a combination of confirmatory and exploratory factor analyses to assess the dimensionality of the ReQoL item sets at the stage of instrument development. The results from study 1 were replicated in the study 2 adding to the robustness of results. We found that a bi-factor model with the positively worded and negatively worded questions as separate factors provided an acceptable fit. The factor loadings were substantially higher on the general factor than on the group factors and the ECV was 78.9% in study 1, and 84.5% in study 2. We have not found commonly agreed threshold for interpreting the ECV, but previous studies have concluded that scales were sufficiently unidimensional if they obtained ECV values in this range [21]. Thus, these results support the assumption of sufficient unidimensionality for IRT analyses [22–24]. Furthermore, the results did not suggest that deviation for perfect unidimensionality is caused by intermingling of positive and negative items which meant that we could consider an intermingled format for the final versions of the ReQoL.

The initial CFA raised questions around the hypothesis of six separate mental health themes identified in earlier work about what mattered to service users with mental health difficulties. This result and subsequent CFA results from a bi-factor model were driven by the high correlation among the themes. From the qualitative work, service users considered the themes separately but the factor analyses suggested that they all amount to one concept of quality of life. To retain the face validity of the ReQoL measure, a decision was taken by the Psychometrics advisory team to select at least one item from each of the themes despite most of the items being highly correlated.

In order to capture the breadth of patient’s journey, the construction of outcome measures requires the inclusion of both positively and negatively worded items. Understandably, there has been a recent focus on framing patients’ experiences positively as exemplified, for example, in the Warwick–Edinburgh mental well-being scale (WEMWBS) [25]. However, patients’ experiences of mental health difficulties are negative. Simply turning a negative item into a positive one does not ensure capturing the same rating. Nor can it be guaranteed that scoring very low on an item phrased as ‘I am feeling happy’ is equivalent to a patient feeling depressed. Such items cannot be viewed as mirror opposites. In addition, while a measure comprising all negative items (for example, Patient Health Questionnaire-9 [26]) might be viewed as being too negative, framing all items as positive could also be viewed by patients as unrealistic and out of touch with the current experiences. Given the bandwidth of patients’ lived experiences, the inclusion of both positive and negative items would appear to be more appropriate for capturing patients’ experiences, an argument used in the development of other measures (for example, Clinical Outcomes in Routine Evaluation [27, 28]).

At the time of development, we searched the literature for guidance on the ordering of positive and negative items. While there was an important literature on positive and negative items in mental health (for example see [26–28]), we could not find any research on how best to order the items. There was no evidence in our study that blocking or intermingling the ordering of the items had an impact on the responses provided by participants. Given this result, on a relatively large item set of 40, we followed the general custom and practice of ordering the items in an intermingled fashion although we used pragmatic rules of not starting and finishing on an extreme negative item and also not having more than three negative items running consecutively. Hence, we were able to incorporate greater flexibility in utilising the intermingled order while also not presenting participants with what might be construed as a short set of negative items followed by a short set of positive items. Our view was that this was a false dichotomy and may appear an artificial separation.

The main limitation of this paper is the fact that participants in study 2 were not randomly assigned to the intermingled and block formats of the ReQoL items. The random assignment of both formats within trust would have made gathering follow-up data needed to assess responsiveness of items reported elsewhere [1] logistically very complicated and prohibitive in terms of resources. However, the results obtained provide robust evidence that the ordering of the items in the questionnaire has not impacted on the questionnaire. This informed the format of the final measures and the main attraction of intermingling the positive and negative items rests in the fact that acquiescence bias can be prevented.

In summary, the factorial structure of the items sets used to construct the ReQoL-10 and ReQoL-20 measures were found to be sufficiently unidimensional as confirmed by the good fit of the bi-factor models and therefore rendering the use of IRT methods to inform further item reduction possible.

## Electronic supplementary material

Below is the link to the electronic supplementary material.

Supplementary material 1 (DOCX 59 KB)
