# Supplementary material for: Exploring the item sets of the Recovering Quality of Life (ReQoL) measures using factor analysis
Source: Qual Life Res. 2018 Dec 21;28(4):1005–15. doi: 10.1007/s11136-018-2091-1 (PMC6439178; doi:10.1007/s11136-018-2091-1)
Supplement: Supplementary file 1 — Supplementary material 1 (DOCX 59 KB) [file 11136_2018_2091_MOESM1_ESM.docx]

**Supplementary online materials**

Table S1 Study 1: Endorsement frequencies, ceiling and floor effects, mean score.

| **Description** | **Variable name** | Level % | | | | | Raw scores | |
| --- | --- | --- | --- | --- | --- | --- | --- | --- |
|  |  | 0 | 1 | 2 | 3 | 4 | Mean | SD |
| I found it difficult to get started with everyday tasks | ACT1 | 17 | 23 | 25 | 19 | 17 | 1.96 | 1.33 |
| I did things that I found rewarding | ACT2P | 14 | 23 | 34 | 20 | 10 | 1.89 | 1.17 |
| I neglected myself | ACT3 | 8 | 13 | 23 | 21 | 35 | 2.61 | 1.30 |
| I avoided things I needed to do | ACT4 | 12 | 20 | 27 | 22 | 19 | 2.17 | 1.28 |
| I enjoyed what I did | ACT5P | 10 | 22 | 30 | 20 | 18 | 2.13 | 1.23 |
| My mental health limited day to day activities | ACT6 | 13 | 18 | 23 | 19 | 26 | 2.27 | 1.37 |
| I found things interesting | ACT7P | 11 | 23 | 32 | 21 | 13 | 2.01 | 1.18 |
| People around me caused me distress | BEL1 | 9 | 15 | 25 | 27 | 24 | 2.44 | 1.24 |
| I felt lonely | BEL2 | 16 | 20 | 24 | 19 | 22 | 2.14 | 1.76 |
| I felt able to trust others | BEL3P | 8 | 21 | 30 | 23 | 18 | 2.23 | 1.20 |
| I felt people did not want to be around me | BEL4 | 9 | 13 | 23 | 20 | 36 | 2.60 | 1.33 |
| I thought people cared about me | BEL5P | 7 | 19 | 30 | 21 | 24 | 2.36 | 1.22 |
| There were people I could turn to for help | BEL6P | 9 | 19 | 24 | 23 | 25 | 2.34 | 1.29 |
| I was able to help others | BEL7P | 10 | 18 | 36 | 22 | 14 | 2.12 | 1.16 |
| I thought nobody cared about me | BEL8 | 10 | 15 | 21 | 16 | 38 | 2.56 | 1.39 |
| I enjoyed being with other people | BEL9P | 9 | 19 | 32 | 20 | 21 | 2.26 | 1.23 |
| I could do the things I wanted to do | CHO1P | 7 | 23 | 31 | 19 | 21 | 2.24 | 1.20 |
| I felt overwhelmed by my problems | CHO2 | 19 | 21 | 22 | 20 | 19 | 1.99 | 1.39 |
| I had the opportunity to do the things I wanted to do | CHO3P | 10 | 23 | 31 | 21 | 16 | 2.11 | 1.21 |
| I felt unable to cope | CHO4 | 12 | 16 | 23 | 21 | 28 | 2.38 | 1.35 |
| I felt in control of my life | CHO5P | 18 | 26 | 24 | 15 | 16 | 1.86 | 1.33 |
| I had choices about what I did | CHO6P | 5 | 11 | 26 | 29 | 29 | 2.66 | 1.15 |
| I felt trapped | CHO7 | 14 | 15 | 20 | 19 | 32 | 2.40 | 1.42 |
| I was able to cope with everyday life | CHO8P | 7 | 19 | 29 | 20 | 25 | 2.38 | 1.24 |
| I felt hopeful about my future | HOP1P | 21 | 23 | 26 | 15 | 15 | 1.81 | 1.33 |
| I felt hopeless | HOP2 | 14 | 17 | 21 | 19 | 29 | 2.34 | 1.40 |
| Everything in my life felt bad | HOP3 | 10 | 13 | 20 | 22 | 35 | 2.57 | 1.35 |
| I thought my life was not worth living | HOP4 | 10 | 11 | 15 | 18 | 46 | 2.79 | 1.37 |
| I believed I could make positive changes | HOP5P | 15 | 25 | 31 | 16 | 13 | 1.87 | 1.23 |
| My life seemed pointless | HOP6 | 14 | 13 | 16 | 15 | 42 | 2.59 | 1.47 |
| I felt like a failure | SEL1 | 18 | 16 | 20 | 17 | 28 | 2.23 | 1.46 |
| I felt confident in myself | SEL2P | 19 | 25 | 27 | 14 | 15 | 1.80 | 1.31 |
| I felt at ease with who I am | SEL3P | 19 | 22 | 25 | 16 | 17 | 1.90 | 1.36 |
| I valued myself as a person | SEL4P | 19 | 23 | 25 | 15 | 18 | 1.91 | 1.36 |
| I disliked myself | SEL5 | 15 | 18 | 19 | 18 | 31 | 2.33 | 1.44 |
| I felt unsure of myself | SEL6 | 14 | 22 | 27 | 22 | 15 | 2.01 | 1.27 |
| I tended to blame myself for bad things that have happened | SEL7 | 19 | 20 | 23 | 17 | 21 | 2.02 | 1.40 |
| I felt confused about who I am | SEL8 | 11 | 12 | 16 | 16 | 45 | 2.72 | 1.41 |
| I felt ok about myself | SEL9P | 14 | 24 | 28 | 17 | 17 | 1.99 | 1.30 |
| I felt calm | WB1P | 7 | 23 | 33 | 20 | 17 | 2.18 | 1.16 |
| I felt safe | WB3P | 6 | 14 | 24 | 22 | 35 | 2.66 | 1.24 |
| I was disturbed by unwanted thoughts and feelings | WB4 | 15 | 21 | 23 | 19 | 22 | 2.12 | 1.37 |
| I felt irritated | WB5 | 14 | 24 | 27 | 22 | 13 | 1.97 | 1.24 |
| I felt angry | WB6 | 9 | 15 | 26 | 27 | 22 | 2.39 | 1.23 |
| I felt relaxed | WB7P | 14 | 27 | 31 | 16 | 12 | 1.85 | 1.20 |
| I felt terrified | WB8 | 7 | 10 | 15 | 18 | 49 | 2.92 | 1.30 |
| I felt everything was an effort | WB9 | 20 | 20 | 25 | 20 | 16 | 1.92 | 1.34 |
| I felt panic | WB10 | 10 | 15 | 20 | 21 | 34 | 2.55 | 1.34 |
| I felt happy | WB11P | 12 | 23 | 32 | 20 | 14 | 2.01 | 1.20 |
| I found it hard to concentrate | WB12 | 19 | 21 | 28 | 18 | 14 | 1.86 | 1.30 |
| I worried too much | WB13 | 26 | 24 | 21 | 17 | 13 | 1.69 | 1.37 |
| I felt anxious | WB14 | 19 | 22 | 22 | 21 | 16 | 1.93 | 1.36 |
| I had problems with my sleep | WB15 | 28 | 19 | 19 | 17 | 17 | 1.78 | 1.45 |
| I had difficulty controlling my worry | WB16 | 15 | 21 | 27 | 23 | 14 | 1.98 | 1.26 |
| I felt tired and worn out | WB17 | 28 | 27 | 23 | 15 | 8 | 1.47 | 1.25 |
| I felt scared | WB18 | 10 | 14 | 19 | 19 | 39 | 2.64 | 1.36 |
| I had feelings of despair | WB19 | 12 | 16 | 21 | 19 | 32 | 2.44 | 1.38 |

Table S2 Study 2: Endorsement frequencies, ceiling and floor effects, mean score.

|  |  | Levels in % | | | | | Raw scores | |
| --- | --- | --- | --- | --- | --- | --- | --- | --- |
|  |  | 0 | 1 | 2 | 3 | 4 | Mean | SD |
| I found it difficult to get started with everyday tasks | ACT1 | 14 | 21 | 25 | 22 | 18 | 2.08 | 1.31 |
| I did things I found rewarding | ACT2P | 14 | 23 | 29 | 19 | 14 | 1.96 | 1.25 |
| I neglected myself | ACT3 | 8 | 13 | 19 | 19 | 40 | 2.70 | 1.34 |
| I avoided things I needed to do | ACT4 | 14 | 20 | 24 | 20 | 22 | 2.17 | 1.34 |
| I enjoyed what I did | ACT5P | 11 | 20 | 30 | 18 | 20 | 2.16 | 1.27 |
| People around me caused me distress | BEL1 | 8 | 15 | 24 | 25 | 28 | 2.50 | 1.26 |
| I felt lonely | BEL2 | 15 | 17 | 20 | 19 | 29 | 2.30 | 1.43 |
| I felt able to trust others | BEL3P | 11 | 20 | 24 | 22 | 23 | 2.24 | 1.31 |
| I felt people did not want to be around me | BEL4 | 11 | 13 | 19 | 17 | 40 | 2.62 | 1.39 |
| I thought people cared about me | BEL5P | 8 | 17 | 25 | 23 | 28 | 2.46 | 1.26 |
| I could do the things I wanted to do | CHO1P | 10 | 24 | 28 | 17 | 20 | 2.14 | 1.27 |
| I felt overwhelmed by my problems | CHO2 | 17 | 18 | 20 | 18 | 26 | 2.18 | 1.44 |
| I had the opportunity to do the things I wanted | CHO3P | 12 | 23 | 28 | 19 | 18 | 2.10 | 1.27 |
| I felt unable to cope | CHO4 | 12 | 16 | 21 | 20 | 31 | 2.43 | 1.38 |
| I felt in control of my life | CHO5P | 20 | 23 | 22 | 16 | 19 | 1.91 | 1.39 |
| I felt hopeful about my future | HOP1P | 17 | 23 | 25 | 16 | 18 | 1.94 | 1.34 |
| I felt hopeless | HOP2 | 12 | 16 | 19 | 18 | 35 | 2.48 | 1.41 |
| Everything in my life felt bad | HOP3 | 10 | 14 | 18 | 19 | 39 | 2.62 | 1.38 |
| I thought my life was not worth living | HOP4 | 9 | 11 | 14 | 15 | 51 | 2.88 | 1.38 |
| I felt like a failure | SEL1 | 17 | 16 | 17 | 17 | 33 | 2.34 | 1.48 |
| I felt confident in myself | SEL2P | 20 | 24 | 24 | 25 | 17 | 1.85 | 1.36 |
| I felt at ease with who I am | SEL3P | 23 | 23 | 23 | 23 | 23 | 2.01 | 1.43 |
| I valued myself as a person | SEL4P | 19 | 22 | 23 | 15 | 22 | 1.99 | 1.41 |
| I disliked myself | SEL5 | 17 | 16 | 17 | 17 | 34 | 2.36 | 1.49 |
| I felt calm | WB1P | 9 | 23 | 30 | 19 | 18 | 2.13 | 1.22 |
| I felt miserable | WB2 | 12 | 20 | 24 | 23 | 21 | 2.22 | 1.30 |
| I felt safe | WB3P | 8 | 15 | 20 | 21 | 36 | 2.62 | 1.32 |
| I was disturbed by unwanted thoughts and feelings | WB4 | 15 | 19 | 21 | 19 | 25 | 2.20 | 1.41 |
| I felt irritated | WB5 | 12 | 22 | 26 | 24 | 16 | 2.11 | 1.25 |
| I felt angry | WB6 | 9 | 15 | 24 | 25 | 26 | 2.45 | 1.27 |
| I felt relaxed | WB7P | 15 | 27 | 26 | 17 | 14 | 1.88 | 1.27 |
| I felt terrified | WB8 | 6 | 9 | 15 | 16 | 53 | 3.02 | 1.26 |
| I felt everything was an effort | WB9 | 18 | 20 | 22 | 21 | 18 | 2.02 | 1.37 |
| I felt panic | WB10 | 9 | 16 | 19 | 17 | 39 | 2.63 | 1.37 |
| I felt happy | WB11P | 14 | 25 | 27 | 18 | 15 | 1.96 | 1.27 |
| I found it hard to concentrate | WB12 | 19 | 21 | 24 | 21 | 16 | 1.93 | 1.34 |
| I worried too much | WB13 | 25 | 24 | 18 | 18 | 16 | 1.76 | 1.41 |
| I felt anxious | WB14 | 21 | 22 | 20 | 20 | 17 | 1.88 | 1.39 |
| I had problems with my sleep | WB15 | 26 | 19 | 17 | 17 | 20 | 1.87 | 1.49 |

*Table S3 Parameter estimates for the two factor and bi-factor original models for Study 1*

|  |  | Two-factor model | | Bi-factor model | | |
| --- | --- | --- | --- | --- | --- | --- |
| **Description** | **Variable name** | Standardised loadings | | Standardised loadings | | |
|  |  | NEG | POS | GLOBAL | NEG | POS |
| I found it difficult to get started with everyday tasks | ACT1 | 0.74 |  | 0.643 | 0.357 |  |
| I did things that I found rewarding | ACT2P |  | 0.749 | 0.715 |  | 0.283 |
| I neglected myself | ACT3 | 0.725 |  | 0.634 | 0.34 |  |
| I avoided things I needed to do | ACT4 | 0.746 |  | 0.647 | 0.365 |  |
| I enjoyed what I did | ACT5P |  | 0.85 | 0.819 |  | 0.277 |
| My mental health limited day to day activities | ACT6 | 0.698 |  | 0.588 | 0.381 |  |
| I found things interesting | ACT7P |  | 0.776 | 0.742 |  | 0.291 |
| People around me caused me distress | BEL1 | 0.647 |  | 0.549 | 0.345 |  |
| I felt lonely | BEL2 | 0.715 |  | 0.614 | 0.363 |  |
| I felt able to trust others | BEL3P |  | 0.659 | 0.61 |  | 0.39 |
| I felt people did not want to be around me | BEL4 | 0.745 |  | 0.649 | 0.356 |  |
| I thought people cared about me | BEL5P |  | 0.655 | 0.597 |  | 0.452 |
| There were people I could turn to for help | BEL6P |  | 0.495 | 0.423 |  | 0.543 |
| I was able to help others | BEL7P |  | 0.525 | 0.478 |  | 0.372 |
| I thought nobody cared about me | BEL8 | 0.783 |  | 0.691 | 0.353 |  |
| I enjoyed being with other people | BEL9P |  | 0.701 | 0.657 |  | 0.356 |
| I could do the things I wanted to do | CHO1P |  | 0.703 | 0.675 |  | 0.247 |
| I felt overwhelmed by my problems | CHO2 | 0.855 |  | 0.716 | 0.475 |  |
| I had the opportunity to do the things I wanted to do | CHO3P |  | 0.715 | 0.675 |  | 0.336 |
| I felt unable to cope | CHO4 | 0.857 |  | 0.725 | 0.461 |  |
| I felt in control of my life | CHO5P |  | 0.856 | 0.849 |  | 0.071 |
| I had choices about what I did | CHO6P |  | 0.519 | 0.481 |  | 0.314 |
| I felt trapped | CHO7 | 0.792 |  | 0.696 | 0.363 |  |
| I was able to cope with everyday life | CHO8P |  | 0.817 | 0.809 |  | 0.086 |
| I felt hopeful about my future | HOP1P |  | 0.831 | 0.818 |  | 0.135 |
| I felt hopeless | HOP2 | 0.876 |  | 0.77 | 0.402 |  |
| Everything in my life felt bad | HOP3 | 0.868 |  | 0.757 | 0.414 |  |
| I thought my life was not worth living | HOP4 | 0.799 |  | 0.693 | 0.391 |  |
| I believed I could make positive changes | HOP5P |  | 0.702 | 0.677 |  | 0.224 |
| My life seemed pointless | HOP6 | 0.895 |  | 0.793 | 0.397 |  |
| I felt like a failure | SEL1 | 0.873 |  | 0.757 | 0.427 |  |
| I felt confident in myself | SEL2P |  | 0.893 | 0.892 |  | 0.027 |
| I felt at ease with who I am | SEL3P |  | 0.885 | 0.888 |  | -0.011 |
| I valued myself as a person | SEL4P |  | 0.848 | 0.841 |  | 0.068 |
| I disliked myself | SEL5 | 0.852 |  | 0.762 | 0.357 |  |
| I felt unsure of myself | SEL6 | 0.73 |  | 0.626 | 0.372 |  |
| I tended to blame myself for bad things that have happened | SEL7 | 0.739 |  | 0.618 | 0.416 |  |
| I felt confused about who I am | SEL8 | 0.813 |  | 0.683 | 0.45 |  |
| I felt ok about myself | SEL9P |  | 0.872 | 0.868 |  | 0.044 |
| I felt calm | WB1P |  | 0.772 | 0.774 |  | -0.008 |
| I felt safe | WB3P |  | 0.742 | 0.733 |  | 0.095 |
| I was disturbed by unwanted thoughts and feelings | WB4 | 0.792 |  | 0.666 | 0.437 |  |
| I felt irritated | WB5 | 0.765 |  | 0.647 | 0.413 |  |
| I felt angry | WB6 | 0.683 |  | 0.564 | 0.404 |  |
| I felt relaxed | WB7P |  | 0.843 | 0.838 |  | 0.059 |
| I felt terrified | WB8 | 0.831 |  | 0.643 | 0.572 |  |
| I felt everything was an effort | WB9 | 0.786 |  | 0.68 | 0.387 |  |
| I felt panic | WB10 | 0.843 |  | 0.671 | 0.542 |  |
| I felt happy | WB11P |  | 0.805 | 0.791 |  | 0.137 |
| I found it hard to concentrate | WB12 | 0.794 |  | 0.679 | 0.41 |  |
| I worried too much | WB13 | 0.78 |  | 0.642 | 0.463 |  |
| I felt anxious | WB14 | 0.859 |  | 0.712 | 0.494 |  |
| I had problems with my sleep | WB15 | 0.596 |  | 0.521 | 0.278 |  |
| I had difficulty controlling my worry | WB16 | 0.666 |  | 0.544 | 0.407 |  |
| I felt tired and worn out | WB17 | 0.686 |  | 0.595 | 0.334 |  |
| I felt scared | WB18 | 0.856 |  | 0.679 | 0.554 |  |
| I had feelings of despair | WB19 | 0.872 |  | 0.743 | 0.457 |  |

*Table S4 Parameter estimates for the two factor and bi-factor original models for Study 2*

|  | | Two-factor model | | Bi-factor model | | |
| --- | --- | --- | --- | --- | --- | --- |
|  | | Standardised loadings | | Standardised loadings | | |
| Description |  | NEG | POS | GLOBAL | NEG | POS |
| I found it difficult to get started with everyday tasks | ACT1 | 0.747 |  | 0.753 | -0.012 |  |
| I did things I found rewarding | ACT2P |  | 0.802 | 0.641 |  | 0.528 |
| I neglected myself | ACT3 | 0.762 |  | 0.761 | 0.056 |  |
| I avoided things I needed to do | ACT4 | 0.783 |  | 0.776 | 0.111 |  |
| I enjoyed what I did | ACT5P |  | 0.865 | 0.718 |  | 0.496 |
| People around me caused me distress | BEL1 | 0.678 |  | 0.65 | 0.259 |  |
| I felt lonely | BEL2 | 0.795 |  | 0.8 | 0.003 |  |
| I felt able to trust others | BEL3P |  | 0.72 | 0.605 |  | 0.39 |
| I felt people did not want to be around me | BEL4 | 0.797 |  | 0.794 | 0.075 |  |
| I thought people cared about me | BEL5P |  | 0.7 | 0.564 |  | 0.456 |
| I could do the things I wanted to do | CHO1P |  | 0.774 | 0.629 |  | 0.484 |
| I felt overwhelmed by my problems | CHO2 | 0.874 |  | 0.861 | 0.155 |  |
| I had the opportunity to do the things I wanted | CHO3P |  | 0.744 | 0.579 |  | 0.535 |
| I felt unable to cope | CHO4 | 0.892 |  | 0.882 | 0.133 |  |
| I felt in control of my life | CHO5P |  | 0.883 | 0.758 |  | 0.433 |
| I felt hopeful about my future | HOP1P |  | 0.782 | 0.647 |  | 0.457 |
| I felt hopeless | HOP2 | 0.899 |  | 0.905 | 0.001 |  |
| Everything in my life felt bad | HOP3 | 0.899 |  | 0.904 | 0.008 |  |
| I thought my life was not worth living | HOP4 | 0.834 |  | 0.84 | -0.005 |  |
| I felt like a failure | SEL1 | 0.888 |  | 0.895 | -0.014 |  |
| I felt confident in myself | SEL2P |  | 0.891 | 0.763 |  | 0.442 |
| I felt at ease with who I am | SEL3P |  | 0.879 | 0.743 |  | 0.467 |
| I valued myself as a person | SEL4P |  | 0.884 | 0.752 |  | 0.456 |
| I disliked myself | SEL5 | 0.87 |  | 0.877 | -0.022 |  |
| I felt calm | WB1P |  | 0.811 | 0.707 |  | 0.361 |
| I felt miserable | WB2 | 0.853 |  | 0.855 | 0.032 |  |
| I felt safe | WB3P |  | 0.748 | 0.646 |  | 0.35 |
| I was disturbed by unwanted thoughts and feelings | WB4 | 0.791 |  | 0.78 | 0.14 |  |
| I felt irritated | WB5 | 0.753 |  | 0.707 | 0.386 |  |
| I felt angry | WB6 | 0.738 |  | 0.688 | 0.405 |  |
| I felt relaxed | WB7P |  | 0.864 | 0.747 |  | 0.409 |
| I felt terrified | WB8 | 0.811 |  | 0.771 | 0.338 |  |
| I felt everything was an effort | WB9 | 0.825 |  | 0.822 | 0.075 |  |
| I felt panic | WB10 | 0.843 |  | 0.796 | 0.382 |  |
| I felt happy | WB11P |  | 0.897 | 0.764 |  | 0.458 |
| I found it hard to concentrate | WB12 | 0.805 |  | 0.792 | 0.153 |  |
| I worried too much | WB13 | 0.807 |  | 0.764 | 0.355 |  |
| I felt anxious | WB14 | 0.845 |  | 0.795 | 0.403 |  |
| I had problems with my sleep | WB15 | 0.655 |  | 0.643 | 0.135 |  |

*Table S3 Parameter estimates for the two factor and bi-factor revised models for Study 1*

|  |  | Two factor model | | Bi-factor model | | |
| --- | --- | --- | --- | --- | --- | --- |
| **Description** | **Variable name** | **NEG** | **POS** | **GLOBAL** | **NEG** | **POS** |
| I found it difficult to get started with everyday tasks | ACT1 | 0.740 |  | 0.638 | 0.353 |  |
| I did things that I found rewarding | ACT2P |  | 0.749 | 0.717 |  | 0.278 |
| I neglected myself | ACT3 | 0.726 |  | 0.632 | 0.350 |  |
| I avoided things I needed to do | ACT4 | 0.747 |  | 0.643 | 0.359 |  |
| I enjoyed what I did | ACT5P |  | 0.850 | 0.820 |  | 0.278 |
| My mental health limited day to day activities | ACT6 | 0.698 |  | 0.586 | 0.392 |  |
| I found things interesting | ACT7P |  | 0.776 | 0.743 |  | 0.292 |
| People around me caused me distress | BEL1 | 0.648 |  | 0.549 | 0.350 |  |
| I felt lonely | BEL2 | 0.716 |  | 0.609 | 0.357 |  |
| I felt able to trust others | BEL3P |  | 0.659 | 0.611 |  | 0.399 |
| I felt people did not want to be around me | BEL4 | 0.746 |  | 0.645 | 0.347 |  |
| I thought people cared about me | BEL5P |  | 0.655 | 0.584 |  | 0.489 |
| There were people I could turn to for help | BEL6P |  | 0.495 | 0.424 |  | 0.549 |
| I was able to help others | BEL7P |  | 0.525 | 0.480 |  | 0.370 |
| I thought nobody cared about me | BEL8 | 0.784 |  | 0.671 | 0.371 |  |
| I enjoyed being with other people | BEL9P |  | 0.701 | 0.658 |  | 0.359 |
| I could do the things I wanted to do | CHO1P |  | 0.703 | 0.677 |  | 0.177 |
| I felt overwhelmed by my problems | CHO2 | 0.856 |  | 0.713 | 0.488 |  |
| I had the opportunity to do the things I wanted to do | CHO3P |  | 0.715 | 0.675 |  | 0.289 |
| I felt unable to cope | CHO4 | 0.858 |  | 0.723 | 0.472 |  |
| I felt in control of my life | CHO5P |  | 0.856 | 0.85 |  | 0.069 |
| I had choices about what I did | CHO6P |  | 0.519 | 0.485 |  | 0.293 |
| I felt trapped | CHO7 | 0.793 |  | 0.695 | 0.370 |  |
| I was able to cope with everyday life | CHO8P |  | 0.817 | 0.81 |  | 0.085 |
| I felt hopeful about my future | HOP1P |  | 0.831 | 0.819 |  | 0.132 |
| I felt hopeless | HOP2 | 0.876 |  | 0.768 | 0.401 |  |
| Everything in my life felt bad | HOP3 | 0.869 |  | 0.756 | 0.423 |  |
| I thought my life was not worth living | HOP4 | 0.800 |  | 0.685 | 0.380 |  |
| I believed I could make positive changes | HOP5P |  | 0.702 | 0.678 |  | 0.226 |
| My life seemed pointless | HOP6 | 0.896 |  | 0.789 | 0.389 |  |
| I felt like a failure | SEL1 | 0.874 |  | 0.756 | 0.419 |  |
| I felt confident in myself | SEL2P |  | 0.893 | 0.892 |  | 0.025 |
| I felt at ease with who I am | SEL3P |  | 0.885 | 0.888 |  | -0.011 |
| I valued myself as a person | SEL4P |  | 0.848 | 0.842 |  | 0.071 |
| I disliked myself | SEL5 | 0.852 |  | 0.761 | 0.365 |  |
| I felt unsure of myself | SEL6 | 0.73 |  | 0.624 | 0.381 |  |
| I tended to blame myself for bad things that have happened | SEL7 | 0.739 |  | 0.613 | 0.405 |  |
| I felt confused about who I am | SEL8 | 0.815 |  | 0.682 | 0.459 |  |
| I felt ok about myself | SEL9P |  | 0.872 | 0.869 |  | 0.048 |
| I felt calm | WB1P |  | 0.772 | 0.775 |  | -0.014 |
| I felt safe | WB3P |  | 0.742 | 0.729 |  | 0.098 |
| I was disturbed by unwanted thoughts and feelings | WB4 | 0.793 |  | 0.664 | 0.448 |  |
| I felt irritated | WB5 | 0.757 |  | 0.647 | 0.394 |  |
| I felt angry | WB6 | 0.671 |  | 0.563 | 0.377 |  |
| I felt relaxed | WB7P |  | 0.843 | 0.839 |  | 0.055 |
| I felt terrified | WB8 | 0.795 |  | 0.651 | 0.479 |  |
| I felt everything was an effort | WB9 | 0.787 |  | 0.678 | 0.397 |  |
| I felt panic | WB10 | 0.834 |  | 0.677 | 0.518 |  |
| I felt happy | WB11P |  | 0.805 | 0.793 |  | 0.132 |
| I found it hard to concentrate | WB12 | 0.795 |  | 0.677 | 0.420 |  |
| I worried too much | WB13 | 0.781 |  | 0.641 | 0.453 |  |
| I felt anxious | WB14 | 0.860 |  | 0.713 | 0.488 |  |
| I had problems with my sleep | WB15 | 0.596 |  | 0.521 | 0.285 |  |
| I had difficulty controlling my worry | WB16 | 0.667 |  | 0.541 | 0.418 |  |
| I felt tired and worn out | WB17 | 0.687 |  | 0.592 | 0.345 |  |
| I felt scared | WB18 | 0.838 |  | 0.692 | 0.494 |  |
| I had feelings of despair | WB19 | 0.873 |  | 0.741 | 0.468 |  |
|  |  |  |  |  |  |  |
| RESIDUAL CORRELATIONS |  |  |  |  |  |  |
| wb5 with wb6 | 0.421 | wb 5 with wb 6 | | 0.414 |  |  |
| wb18 with wb8 | 0.587 | wb18 with wb8 | | 0.560 |  |  |
| wb8 with wb10 | 0.463 | wb8 with wb10 | | 0.420 |  |  |
|  |  | sel1 with sel7 | | 0.398 |  |  |
|  |  | hop4 with hop6 | | 0.478 |  |  |
|  |  | bel8 with bel4 | | 0.420 |  |  |
|  |  | bel8 with bel5p | | 0.571 |  |  |
|  |  | act4 with act1 | | 0.364 |  |  |
|  |  | cho1p with cho3p | | 0.312 |  |  |
|  |  | hop2 with bel2 | | 0.367 |  |  |
|  |  | wb13 and wb14 | | 0.284 |  |  |
|  |  | wb8 with wb3p | | 0.374 |  |  |
